# Bullying victimisation in adolescence: prevalence and inequalities by gender, socioeconomic status and academic performance across 71 countries

**DOI:** 10.1016/j.eclinm.2021.101142

**Published:** 2021-10-11

**Authors:** Mariko Hosozawa, David Bann, Elian Fink, Esme Elsden, Sachiko Baba, Hiroyasu Iso, Praveetha Patalay

**Affiliations:** a Institute for Global Health Policy Research, Bureau of International Health Cooperation, National Center for Global Health and Medicine, Japan; b Deparetment of Pediatrics, Juntendo University, Japan; c Department of Epidemiology and Public health, UCL, UK; d Centre for Longitudinal Studies, Institute of Social Research, UCL, UK; e School of Psychology, University of Sussex, UK; f Institute of Epidemiology and Public Health, UCL, UK; g Bioethics and Public Policy, Department of Social Medicine, Osaka University Graduate School of Medicine, Japan; h Public Health, Department of Social Medicine, Osaka University Graduate School of Medicine, Japan; i MRC Unit for Lifelong Health and Ageing, UCL, UK

## Abstract

**Background:**

Bullying victimisation is of global importance due to its long-term negative consequences. We examined the prevalence of victimisation and its inequalities in 15-year-olds across 71 countries.

**Methods:**

Data were from the Programme for International Student Assessment (March-August 2018). Students reported frequencies of relational, physical, and verbal victimisation during the last 12 months, which were analysed separately and combined into a total score. Prevalence of frequent victimisation (> a few times a month) was estimated, followed by mean differences in total score by gender, wealth and academic performance quintiles in each country. Meta-analyses were used to examine country differences.

**Findings:**

Of 421,437 students included, 113,602 (30·4%) experienced frequent victimisation, yet this varied by country—from 9·3% (Korea) to 64·8% (Philippines). Verbal and relational victimisation were more frequent (21·4%, 20.9%, respectively) than physical victimisation (15·2%). On average, boys (vs girls +0·23SD, 95%CI: 0·22–0·24), students from the lowest wealth (vs highest +0·09SD, 0·08–0·10) and with lowest academic performance (vs highest +0·49SD, 0·48–0·50) had higher scores. However, there was substantial between-country heterogeneity in these associations (I^2^=85%–98%). Similar results were observed for subtypes of victimisation—except relational victimisation, where gender inequalities were smaller.

**Interpretation:**

Globally, bullying victimisation was high, although the size, predominant subtype and strength of associations with risk factors varied by country. The large cross-country differences observed require further replication and empirical explanation, and suggest the need to and the large scope for reducing bullying victimisation and its inequity in the future.

**Funding:**

Japan Foundation for Pediatric Research

**Research in context**

**Evidence before this study**

We searched PubMed for studies published up to December 1, 2020, with the following terms ("bulli*" or "bully*" or "peer victimisation") AND ("cross-country" or "cross-national" or "multi-country"). We also searched for references cited in relevant publications. Existing cross-national studies and official reports of large-scale cross-country studies report a considerable difference in the prevalence of bullying victimisation (hereafter victimisation) across countries. Prior studies have mostly focused on overall victimisation; however, the subtypes (relational, physical, verbal) and the strength of association with risk factors may differ by country.

**Added value of this study**

Using a nationally-representative data of 15-year-olds across 71 high and middle-income countries, the overall prevalence of frequent victimisation (> a few times a month) was 30·4%, with substantial between-country heterogeneity (range 9·3 to 64·8). Relational and verbal victimisation were more common compared to physical victimisation; however, large between-country heterogeneity was also observed in these prevalences. On average, boys, students with the lowest family wealth and lowest academic performance were more likely to be victimised; the magnitude of inequalities by socioeconomic position were smaller and not evident in some Asian or European A countries.

**Implications of all the available evidence**

The high prevalence of victimisation at the global level in the previous and current studies highlight the need for preventive interventions globally. The substantial between-country heterogeneity in its prevalence, predominant subtype and risk factors highlight the context-dependence of victimisation and hence it's potential modifiability. Advancing our understanding of the heterogeneity observed, accounting for the predominant types in that country, and learning from countries where the prevalence and inequalities in victimisation are lower might prove beneficial in identifying successful strategies to reduce its prevalence and the multiple forms of inequalities observed.

Alt-text: Unlabelled box

## 1 Introduction

Bullying victimisation (hereafter victimisation), the repeated and targeted experience of aggressive behaviour in the context of an imbalance of power [1], is of increasing public health concern given its links with poor subsequent mental health and other adverse health/social outcomes [2]. While bullying is widespread during adolescence, cross-national studies have reported sizable variability in its prevalence; for instance, within Europe, only 5% of youth in Sweden report experiencing victimisation, while 20% report experiencing victimisation in Lithuania [3]. The current study examines the prevalence of victimisation and its subtypes (relational, physical and verbal victimisation); and investigates gender, socioeconomic and academic performance based inequalities in victimisation across 71 countries.

Victimisation can take various forms, including physical (e.g., hitting, pushing, damaging property), verbal (e.g., name-calling, teasing, intimidation) and relational (manipulation of social relationships, e.g., gossip, spreading rumours) [4]. Importantly, different subtypes of victimisation have different implications for psychological health, and anti-bullying intervention programs are differentially effective across different types of victimisation [5]. However, most population-based and cross-national investigations of victimisation rely on a single-item measure of overall victimisation, precluding an examination of variability in different subtypes of victimisation. One exception is the work by Craig and colleagues [6], who found considerable differences in the prevalence of victimisation subtypes across six high- and middle-income western countries.

Alongside a focus on overall victimisation, existing cross-national victimisation research largely focuses on countries in Europe and North America [3,4,[6], [7], [8]]. This is particularly problematic as victimisation and its subtypes may have different levels of social acceptability and be rooted in different social norms in different countries. Thus, further work is required to document cross-country differences in victimisation prevalence and the forms this takes.

In addition to understanding cross-national differences in prevalence, it is also important to understand differences and similarities in the predictors of victimisation, with implications for the identification of high-risk groups and anti-bullying intervention programs. Existing evidence has tended to focus on easily measured demographic inequalities in victimisation, for instance, age [6,7,9] and gender [6,9,10]. However, inequalities in victimisation are predicted by a range of factors, including sociodemographic factors such as socioeconomic position [8,11,12], and non-demographic factors such as academic performance [13]. Whether these factors are differentially associated with victimisation across different countries is uncertain.

It is likely, given the large variation in sociodemographic inequalities (e.g., gender roles, socioeconomic inequality) across countries, that sociodemographic predictors of different subtypes of victimisation have country- or region-specific patterns. For example, while there is consistent evidence that socioeconomic disadvantage is associated with greater victimisation in high-income countries [8,11], the magnitude of this association across different middle or low-income countries is unknown. It is also unclear if the impact of socioeconomic disadvantage differs by victimisation subtype. Additionally, the association between victimisation and academic performance may differ as a function of both the subtype of victimisation and nationality. For instance, there may be a weaker association between lower academic performance and victimisation risk in high-income White-majority countries compared with Asian countries due to the different values placed on school performance across these countries [13].

Using data from the Programme for International Student Assessment (PISA) 2018, the current study aims to; (i) examine the prevalence of victimisation and subtypes of bullying (relational, physical and verbal) across 71 high and middle-income countries, and (ii) investigate the variability in victimisation cross-nationally as a function of inequalities in gender, socioeconomic position (wealth) and academic performance. The current study builds upon the descriptive results in the PISA 2018 report [14] by examining differences in the rates and distributions of victimisation (both overall and by well-established subtypes that have been previously understudied) in adolescents across a broad range of countries with high-quality, representative data and investigating a range of inequalities cross-nationally to help provide important insights into the contextual factors relevant to victimisation. Examining how these might differentially operate in different cultures and contexts is relevant for understanding aetiology and supporting research and prevention efforts that consider country-specific factors while learning from contexts where prevalences and inequalities are lower.

## 2 Methods

### 2.1 Data source

The PISA is conducted by the Organisation for Economic Co-operation and Development (OECD) in over 70 member and non-member nations and economies [15]. PISA aims to draw a representative sample of in-school pupils in each country aged between 15 years and 3-months and 16 years and 2-months at the time of assessment. It has a two-stage probabilistic, stratified and clustered survey design. First, schools are stratified and then randomly selected with probability proportional to size (within a minimum of 150 schools from each country). All countries must ensure they meet the OECD's response rate of 85% for schools and 80% for pupils to be included in the study—or, in the case of Portugal, have demonstrated little response bias [15]. PISA 2018 was conducted between March to August 2018, and 612,004 students participated, representing about 31 million students in the schools of the 82 participating countries, economies or regions. To aid comparison, we restricted our analyses to 71 countries with available exposure and outcome data: four countries (Cyprus, Israel, Lebanon, North Macedonia) were excluded due to missing bullying data and two countries (Spain and Vietnam) were not made available due to suboptimal response behaviours or technical issues affecting the comparability of the academic performance data; additional subsamples (‘economies’ and ‘regions’) were not included given concerns about national representation (See Figure S1 for sample selection flow chart). This left 526,161 students in eligible countries. We grouped countries into five categories based on the World Health Organization (WHO) classification (https://www.who.int/choice/demography/) and geographical status. Our grouping was as follows: (1) East Mediterranean; (2) South-East Asian & Western Pacific (hereafter referred to as SE Asian + Pacific); (3) Americas (4) Europe A; and (5) Europe B/C. The approval for the collection of the data was obtained from the PISA Governing Board composed of representatives of OECD Members and PISA Associates. In each participating country or economy, school staff, students and parents were informed of the nature of the assessment and its use and consent provided. This study follows the Strengthening the Reporting of Observational Studies in Epidemiology (STROBE) guideline in reporting of the results.

### 2.2 Outcome: Victimisation

The experience of victimisation was assessed by the six questions assessing the frequency of being victimised in different ways corresponding to the relational, physical and verbal subtypes of victimisation (see Supplement for details). ‘During the past 12 months, how often have you had the following experiences in school? (Some experiences can also happen in social media.)’

1 Other students left me out of things on purpose.
2 Other students made fun of me.
3 I was threatened by other students.
4 Other students took away or destroyed things that belonged to me.
5 I got hit or pushed around by other students.
6 Other students spread nasty rumours about me.

Frequency was assessed on a 4-point scale (1= never or almost never, 2 = a few times a year, 3 = a few times a month, and 4 = once a week or more). To quantify the prevalence of overall victimisation, we first created a dichotomised variable defined as being victimised more than a few times a month in either of the six victimisation questions. This definition was used to aid comparability with other large-scale cross-country studies [6,16]. We also created a dichotomised variable representing the prevalence of victimisation by each subtype based on the definition used in PISA reports [14].

The total victimised score was created by summing responses to all six items (range 6–24, higher scores indicate more frequent victimisation). The score had adequate internal consistency in all the countries included in the study (Cronbach's alpha > 0·8). Furthermore, scores for each subtype (i.e. relational, physical and verbal) were created by summing responses to the two questions related to each subtype (range 2–8). To avoid bias due to listwise deletion, participants were included in the study if they responded to at least five of the six victimisation questions; one missing bullying item (missing in 2·8%) was imputed using a person-mean score. The imputed results were broadly similar to those obtained using observed cases and therefore, the imputed results are presented here. To aid interpretation, all the bulling-victimisation scores were converted into standardised z-scores based on the whole PISA population to aid comparability between different outcomes; findings were unchanged when using raw scores.

### 2.3 Correlates: Gender, wealth and academic performance

Gender (male/female) was coded based on the student's self-report. Wealth was measured by reported family wealth possessions, a continuous variable estimated using the OECD's item response theory scaling [17] using twelve standardised questions on possessions and characteristics of the home. Country-specific quintiles of the continuous wealth variable were calculated for use in our analyses.

Academic performance was measured principally via computer-based tests covering three academic domains (mathematics, reading and science literacy) [18]. Participants took a random subsample of test questions and ten ‘plausible values’ for each subject area was provided by the survey organiser to estimate the student's proficiency in that subject. Plausible values from each academic domain were highly correlated. Our confirmatory factor analysis (CFA) showed that plausible values from all three domains contribute to one latent construct of overall academic performance, which explained 86% of the variance. We created an overall academic performance score from predicted values of the CFA, which were divided into quintiles within each country.

### 2.4 Statistical Analyses

We first conducted a sample bias analysis using Chi-squared tests to investigate whether the demographic characteristics of students excluded from our analytic sample (due to missing exposure or outcome data) differed from those included. We then examined correlations between our victimisation scores. A descriptive analysis of our variables was also conducted. Mean differences for outcomes by exposures (i.e. gender, wealth and academic performance quintiles) were estimated for each country. To compare the magnitude of inequality by our exposures across countries, we plotted the mean victimised scores; 1) by gender, 2) for the highest and lowest wealth quintiles, and 3) for the highest and lowest academic performance quintile for each country. Meta-analyses using random effects models were used to formally assess heterogeneity in the associations between gender, wealth and academic performance inequality with our outcome across countries. The I^2^ statistic was calculated to quantify the percentage of variation across nations due to heterogeneity rather than chance [19]. All analyses were performed using Stata V15.0. All models used Balanced Repeated Replication (BRR) weights provided to adjust for the complex survey design.

### 2.5 Role of the funding source

The funders of the study had no role in the study design, data collection, data analysis, data interpretation, or writing of the report. The corresponding author had full access to all the data in the study, and all the authors accept final responsibility for the decision to submit for publication.

## 3 Results

Of the 526,161 students from eligible countries, 421,437 (49·5% boys) were included in the final sample—with participants excluded due to missing academic performance, wealth, or victimisation data. Students who were excluded were more likely to be boys, from lower wealth backgrounds and lower academic performance scores (*p* < 0·0001 for all comparisons; Table S1). All victimisation subtypes were moderately positively correlated (Table S2). The demographic characteristics of our study participants by country and region are shown in Table S3.

### 3.1 Prevalence of victimisation

**Figure 1:**
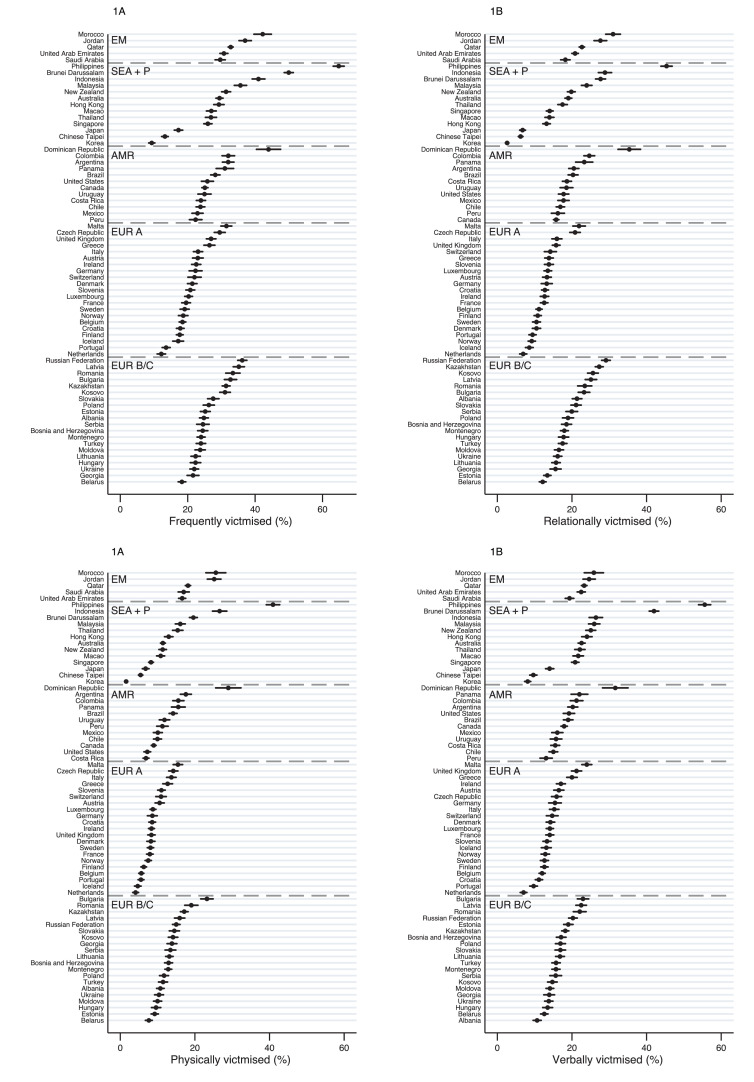
**Prevalence of overall and subtypes of victimisation, by country.** EM = Eastern Mediterranean; SEA+P = South East Asia & Pacific; AMR = Americas; EUR A = Europe A; EUR B/C = Europe B/C. The weighted prevalence of overall and subtypes of victimisation, by country, defined as experiencing victimisation for more than a few times a month during the last 12 months. Error bars show 95% CIs.

The prevalence of victimisation, for overall and each subtype, by country is shown in Figure 1; descriptive statistics across countries and by regions are shown in Table 1. In our sample, 113,602 (30·4%) reported any type of victimisation more than a few times a month during the last 12 months (‘frequent victimised’). There was a substantial between-country difference in this prevalence ranging from 9·3% in the Republic of Korea (hereafter Korea) to 64·8% in the Philippines).

**Table 1:** Descriptive statistics of victimisation prevalence and scores, by country

Region	Country	Total N	Prevalence								Mean victimised scores							
			Frequently victimised^a^		Relationally victimised^b^		Physically victimised^b^		Verbally victimised^b^		Total victimised score		Relationally victimised score		Physically victimised score		Verbally victimised score	
			n	%	n	%	n	%	n	%	Mean	SD	Mean	SD	Mean	SD	Mean	SD
Eastern Mediterranean	Jordan	7,929	2,873	37·1	2,133	27·6	1,920	25·2	1,893	24·6	0·31	1·22	0·23	1·11	0·40	1·30	0·17	1·14
	Morocco	2,455	1,039	42·2	766	31·0	633	25·6	638	25·9	0·41	1·13	0·37	1·11	0·43	1·23	0·26	1·09
	Qatar	11,557	3,757	32·8	2,601	22·7	2,074	18·1	2,666	23·3	0·22	1·12	0·17	1·07	0·19	1·14	0·18	1·08
	Saudi Arabia	5,290	1,567	29·6	972	18·2	892	17·0	1,018	19·4	0·02	1·00	-0·06	0·93	0·09	1·08	0·01	0·99
	United Arab Emirates	17,124	5,497	30·7	3,748	20·9	3,039	16·6	4,034	22·5	0·16	1·13	0·11	1·07	0·14	1·14	0·15	1·10
	Sub-total	44,355	14,733	33·9	10,220	23·1	8,558	20·3	10,249	22·1	0·18	1·09	0·11	1·04	0·23	1·17	0·11	1·06
SE Asian + Pacific	Australia	10,640	3,194	29·4	2,086	19·0	1,257	11·4	2,435	22·6	0·12	1·03	0·11	1·03	-0·02	0·96	0·19	1·08
	Brunei Darussalam	4,739	2,367	50·0	1,309	27·7	927	19·6	1,989	42·0	0·50	1·09	0·34	1·06	0·23	1·10	0·73	1·27
	Chinese Taipei	7,067	914	13·2	432	6·3	380	5·5	666	9·7	-0·38	0·63	-0·40	0·67	-0·30	0·57	-0·34	0·68
	Hong Kong	5,597	1,646	29·3	727	13·2	724	13·0	1,356	24·0	-0·02	0·97	-0·14	0·97	-0·02	0·99	0·07	1·00
	Indonesia	11,604	4,591	41·0	3,181	28·9	2,870	26·6	2,907	26·4	0·39	1·20	0·32	1·13	0·46	1·27	0·24	1·15
	Japan	5,946	1,019	17·3	398	6·8	402	6·8	825	14·0	-0·32	0·72	-0·38	0·71	-0·26	0·72	-0·25	0·80
	Korea	6,593	608	9·3	165	2·6	105	1·6	533	8·1	-0·52	0·45	-0·54	0·46	-0·45	0·38	-0·43	0·62
	Macao	3,757	1,015	27·0	525	14·0	407	10·8	815	21·7	-0·01	0·91	-0·08	0·88	-0·07	0·88	0·09	1·01
	Malaysia	5,805	2,069	35·7	1,389	24·0	913	16·1	1,510	26·0	0·21	1·02	0·23	1·04	0·08	1·00	0·21	1·04
	New Zealand	5,050	1,586	31·4	1,005	19·8	565	11·4	1,263	25·0	0·16	1·02	0·15	1·04	-0·01	0·94	0·26	1·10
	Philippines	6,281	4,071	64·8	2,847	45·4	2,562	40·9	3,495	55·6	1·15	1·31	0·84	1·22	0·98	1·38	1·22	1·33
	Singapore	6,417	1,677	26·0	917	14·1	541	8·2	1,344	20·9	0·01	0·89	0·00	0·93	-0·11	0·83	0·09	0·95
	Thailand	8,351	2,208	26·9	1,435	17·5	1,195	15·4	1,795	22·1	0·10	1·12	0·02	1·04	0·10	1·08	0·13	1·08
	Sub-total	87,847	26,965	37·1	16,416	24·7	12,848	22·1	20,933	27·3	0·30	1·20	0·20	1·12	0·31	1·22	0·26	1·19
Americas	Argentina	7,809	2,386	32·0	1,539	20·5	1,266	17·6	1,512	20·2	0·09	1·02	0·09	1·07	0·08	1·03	0·05	1·03
	Brazil	6,475	1,805	28·2	1,306	20·3	883	14·2	1,216	19·0	0·07	1·05	0·09	1·05	0·01	1·00	0·04	1·04
	Canada	18,998	5,028	25·1	3,277	15·8	1,834	9·0	3,630	17·9	-0·05	0·91	-0·03	0·94	-0·14	0·84	0·00	0·96
	Chile	5,037	1,166	23·8	814	16·9	474	10·0	742	15·0	-0·07	0·94	-0·01	0·96	-0·09	0·89	-0·10	0·91
	Colombia	5,238	1,618	32·1	1,249	24·7	774	15·6	1,063	21·2	0·12	1·09	0·17	1·08	0·06	1·08	0·06	1·02
	Costa Rica	6,295	1,495	23·9	1,186	18·7	433	6·9	950	15·5	-0·09	0·92	0·03	1·02	-0·22	0·79	-0·07	0·97
	Dominican Republic	1,454	631	44·0	509	35·4	420	29·0	456	31·6	0·56	1·35	0·50	1·28	0·50	1·34	0·45	1·27
	Mexico	3,886	870	22·9	663	17·7	381	10·1	609	16·1	-0·07	0·95	-0·03	0·99	-0·10	0·91	-0·08	0·91
	Panama	1,778	566	31·0	417	23·3	288	15·6	407	22·0	0·11	1·12	0·11	1·07	0·07	1·10	0·09	1·10
	Peru	2,180	496	22·4	361	16·2	252	11·3	285	13·1	-0·10	0·91	-0·05	0·93	-0·05	0·94	-0·18	0·86
	United States	4,573	1,189	25·9	829	17·8	343	7·2	877	19·2	-0·05	0·88	0·01	0·96	-0·20	0·76	0·01	0·96
	Uruguay	2,955	764	25·0	569	18·5	374	11·8	485	15·7	-0·02	0·97	0·01	0·99	-0·04	0·95	-0·05	0·93
	Sub-total	66,678	18,014	26·6	12,719	18·7	7,722	10·2	12,232	18·7	-0·01	0·95	0·03	0·99	-0·11	0·88	0·00	0·98
Europe A	Austria	5,329	1,214	23·0	706	13·3	555	10·6	871	16·5	-0·10	0·89	-0·15	0·91	-0·09	0·91	-0·07	0·88
	Belgium	7,249	1,316	18·5	788	11·2	398	5·6	857	12·0	-0·21	0·72	-0·18	0·82	-0·25	0·67	-0·16	0·78
	Croatia	5,529	997	17·8	715	12·8	480	8·6	627	11·2	-0·19	0·89	-0·18	0·91	-0·15	0·87	-0·20	0·87
	Czech Republic	6,184	1,707	29·5	1,192	20·8	805	14·2	913	15·9	0·02	0·98	0·07	1·02	0·07	1·04	-0·09	0·94
	Denmark	6,046	1,337	21·4	673	10·5	554	8·2	884	14·2	-0·12	0·74	-0·15	0·80	-0·11	0·79	-0·11	0·78
	Finland	5,047	892	17·6	547	10·8	318	6·3	640	12·6	-0·18	0·80	-0·17	0·85	-0·22	0·73	-0·12	0·85
	France	4,762	1,002	19·5	649	12·6	426	7·9	720	14·1	-0·17	0·85	-0·17	0·89	-0·20	0·80	-0·12	0·88
	Germany	2,339	515	22·3	303	13·3	201	8·6	361	15·5	-0·13	0·80	-0·15	0·85	-0·15	0·80	-0·08	0·83
	Greece	5,548	1,449	26·4	752	13·9	691	12·7	1,097	20·0	-0·08	0·95	-0·16	0·93	-0·05	0·98	-0·01	0·94
	Iceland	2,451	424	17·2	212	8·6	118	4·7	325	13·1	-0·30	0·74	-0·33	0·76	-0·30	0·67	-0·19	0·84
	Ireland	4,535	1,028	22·5	578	12·6	381	8·4	775	17·0	-0·06	0·87	-0·08	0·89	-0·11	0·82	0·01	0·95
	Italy	8,755	2,043	23·1	1,421	16·0	1,223	13·7	1,350	15·2	-0·08	0·98	-0·07	0·97	-0·03	1·01	-0·13	0·93
	Luxembourg	4,582	926	20·3	618	13·5	399	8·8	644	14·1	-0·13	0·88	-0·12	0·92	-0·15	0·84	-0·10	0·88
	Malta	2,789	873	31·5	608	21·9	428	15·4	664	24·0	0·20	1·12	0·16	1·09	0·13	1·11	0·21	1·09
	Netherlands	3,621	451	12·2	260	6·9	160	4·1	260	7·0	-0·34	0·59	-0·33	0·68	-0·29	0·60	-0·32	0·61
	Norway	5,294	992	18·7	496	9·2	400	7·5	681	12·9	-0·20	0·80	-0·25	0·81	-0·15	0·79	-0·17	0·83
	Portugal	4,898	677	13·6	486	9·4	287	5·6	481	9·7	-0·30	0·76	-0·28	0·78	-0·27	0·70	-0·26	0·77
	Slovenia	5,001	1,109	20·8	742	13·8	617	11·0	733	13·3	-0·11	0·92	-0·10	0·94	-0·07	0·92	-0·15	0·88
	Sweden	4,805	922	19·1	501	10·5	391	8·1	614	12·6	-0·18	0·81	-0·21	0·85	-0·13	0·82	-0·18	0·83
	Switzerland	3,598	808	22·0	537	14·2	408	10·9	547	14·7	-0·08	0·91	-0·11	0·91	-0·06	0·91	-0·06	0·88
	United Kingdom	12,215	3,257	27·0	1,920	15·8	1,043	8·4	2,572	21·2	0·00	0·92	0·01	0·97	-0·16	0·81	0·11	1·01
	Sub-total	110,577	23,939	21·8	14,704	13·4	10,283	9·0	16,616	15·4	-0·12	0·87	-0·12	0·90	-0·14	0·84	-0·09	0·90
Europe B/ C	Albania	5,986	1,505	24·8	1,293	21·4	660	10·7	652	10·7	-0·11	0·98	0·01	0·98	-0·07	1·00	-0·27	0·90
	Belarus	5,391	992	18·3	654	12·2	408	7·6	683	12·6	-0·20	0·83	-0·16	0·86	-0·20	0·78	-0·20	0·82
	Bosnia and Herzegovina	5,414	1,332	24·5	1,009	18·6	712	12·9	934	17·1	-0·04	1·05	-0·04	1·02	-0·04	1·03	-0·06	1·01
	Bulgaria	3,741	1,221	32·7	874	23·2	861	23·2	854	23·0	0·27	1·23	0·16	1·16	0·36	1·27	0·17	1·14
	Estonia	4,826	1,216	25·2	658	13·4	453	9·2	920	19·0	-0·09	0·86	-0·13	0·87	-0·12	0·86	-0·01	0·92
	Georgia	4,369	955	21·6	682	15·6	604	13·8	615	13·9	-0·11	1·02	-0·13	1·00	-0·01	1·03	-0·18	0·93
	Hungary	4,293	937	22·3	742	17·8	396	9·6	567	13·5	-0·12	0·94	-0·01	1·01	-0·14	0·91	-0·19	0·89
	Kazakhstan	15,729	1,375	31·4	1,140	27·3	662	17·1	673	18·2	0·11	1·20	0·16	1·12	0·12	1·19	-0·02	1·10
	Kosovo	4,346	4,651	31·1	4,022	25·6	2,377	14·1	2,634	14·8	0·05	1·01	0·15	0·98	0·07	1·07	-0·12	0·96
	Latvia	4,542	1,615	35·2	1,147	25·1	746	15·9	1,036	22·5	0·23	1·01	0·26	1·02	0·16	1·05	0·16	0·99
	Lithuania	5,540	1,279	22·4	902	15·7	762	13·1	962	16·8	-0·02	1·05	-0·06	0·98	0·00	1·04	-0·02	1·00
	Moldova	4,889	1,156	23·7	796	16·5	490	10·0	694	14·1	-0·04	0·85	-0·01	0·87	-0·06	0·85	-0·07	0·88
	Montenegro	5,525	1,313	24·0	984	18·0	705	12·9	871	15·7	-0·04	1·06	-0·03	1·02	-0·02	1·05	-0·08	1·01
	Poland	5,062	1,327	26·3	968	18·9	592	11·7	852	16·9	0·02	0·96	0·07	0·98	-0·04	0·96	-0·02	0·95
	Romania	4,485	1,496	33·4	1,056	23·4	843	19·0	990	22·1	0·22	1·06	0·19	1·02	0·21	1·08	0·17	1·05
	Russian Federation	6,463	2,386	36·2	1,932	29·1	974	15·0	1,334	20·3	0·16	1·09	0·27	1·09	0·06	1·10	0·06	1·06
	Serbia	4,866	1,182	24·5	964	20·0	648	13·4	758	15·7	-0·04	1·07	0·00	1·06	-0·02	1·06	-0·11	1·00
	Slovak Republic	4,834	1,319	27·6	1,005	21·1	697	14·5	809	16·9	0·07	1·03	0·12	1·04	0·06	1·05	-0·03	0·97
	Turkey	6,627	1,584	23·9	1,160	17·5	759	11·5	1,047	15·7	-0·07	1·00	-0·05	1·00	-0·07	0·98	-0·10	0·96
	Ukraine	5,052	1,110	22·0	819	16·2	522	10·4	696	13·8	-0·10	0·90	-0·03	0·93	-0·11	0·89	-0·15	0·89
	Sub-total	111,980	29,951	28·9	22,807	22·3	14,871	13·3	18,581	17·5	0·04	1·04	0·10	1·04	0·00	1·04	-0·03	1·00
	Overall	421,437	113,602	30·4	76,866	20·9	54,282	15·2	78,611	21·4	0·10	1·07	0·09	1·04	0·07	1·06	0·08	1·06

Unweighted numbers, weighted percentages and means (in z-scores) are shown. SD = standard deviation.

a Defined as experiencing any type of victimisation for more than a few times a month during the last 12 months.

b Defined as experiencing the specified type of victimisation for more than a few times a month during the last 12 months.

Analysis by subtype of victimisation showed that, overall, relational (n = 76,866, 20·9%) and verbal (78,611, 21·4%) victimisation was more common at a similar level, compared with physical victimisation (54,282, 15·2%, Table 1). Country differences were driven by different subtypes of victimisation. For example, all three subtypes showed similar prevalence in some countries, which was due to the higher rates of physical victimisation (e.g., Jordan, Indonesia and Bulgaria). Verbal victimisation was prominently high in some SE Asian countries such as Japan and Korea, where the prevalence of verbal victimisation was more than double of the other subtypes. In contrast, relational victimisation was the predominant subtype in some European B/C countries (e.g., Albania, Kazakhstan, Kosovo and Russian Federation).

### 3.2 Inequalities by gender

**Figure 2:**
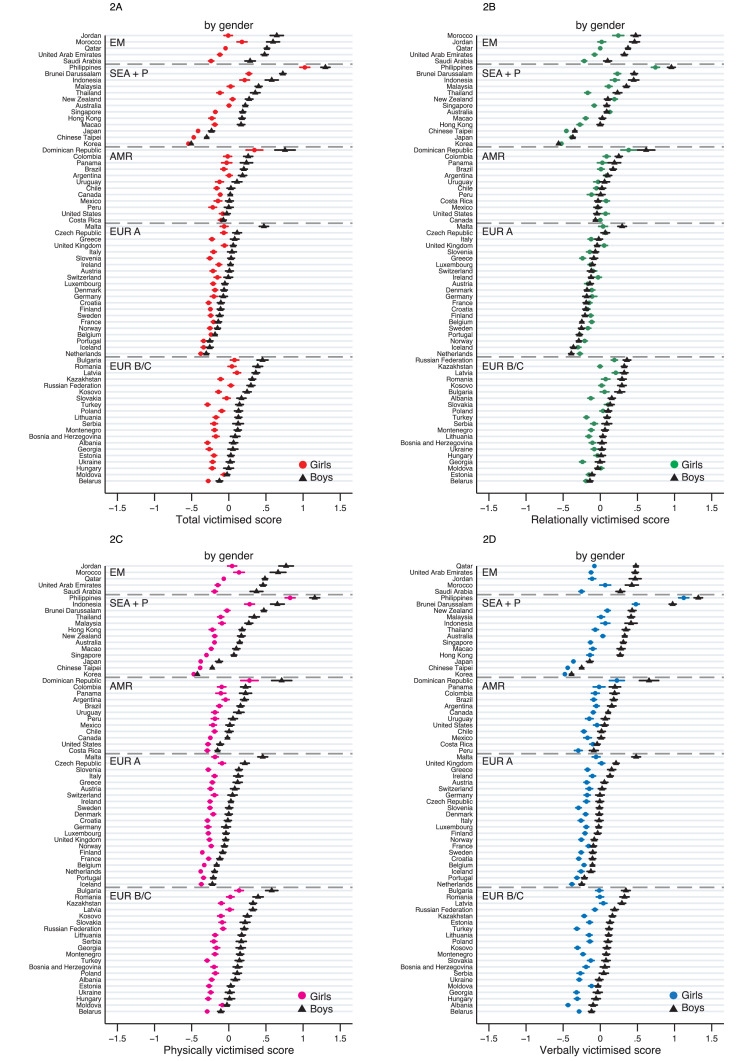
**Inequalities by gender for victimisation scores, by country.** EM = Eastern Mediterranean; SEA+P = South East Asia & Pacific; AMR = Americas; EUR A = Europe A; EUR B/C = Europe B/C. Weighted means (in z-scores) for the total and subtypes of victimisation scores, by gender, are plotted for each country. Error bars show 95% CIs.

The magnitude of inequalities by gender for each country's total victimised score is plotted in Figure 2 and summarised using meta-analysis in Table 2. Overall, boys were more likely to be victimised than girls (boys vs girls 0·23SD, 95%CI 0·22–0·24). However, there was evidence for heterogeneity between countries in the size of the inequality by gender (I^2^ = 97·6). The largest gender inequality in the total victimised score across countries was found in Jordan (0·65, 0·60–0·71), followed by the United Arab Emirates (0·60, 0·57–0·64) while no gender inequality was observed in Costa Rica (0·03, -0·02–0·07) and Moldova (0·04, -0·01–0·09).

**Table 2:** Overall estimate of inequalities for *the total victimised score* by gender, wealth and academic performance, by country

Region	Country	Gender (boys-girls)				Wealth (lowest-highest quintile)				Academic performance (lowest-highest quintile)			
		Mean Difference	95%CI		*I^2^*	Mean Difference	95%CI		*I^2^*	Mean Difference	95%CI		*I^2^*
Eastern Mediterranean	Jordan	0·65	0·60	0·71		0·32	0·23	0·40		0·83	0·75	0·92	
	Morocco	0·42	0·33	0·51		-0·02	-0·17	0·13		0·74	0·60	0·87	
	Qatar	0·56	0·52	0·60		0·23	0·16	0·30		0·87	0·80	0·94	
	Saudi Arabia	0·53	0·47	0·58		0·18	0·09	0·27		0·62	0·53	0·71	
	United Arab Emirates	0·60	0·57	0·64		0·12	0·07	0·18		0·84	0·78	0·90	
	Sub-total	0·58	0·56	0·60	85·8	0·18	0·15	0·22	82·3	0·81	0·77	0·84	82·3
SE Asian + Pacific	Australia	0·22	0·18	0·26		0·07	0·01	0·14		0·52	0·45	0·58	
	Brunei Darussalam	0·46	0·40	0·52		0·39	0·28	0·49		1·19	1·10	1·29	
	Chinese Taipei	0·17	0·15	0·20		0·05	0·01	0·10		0·15	0·10	0·20	
	Hong Kong	0·41	0·36	0·46		0·04	-0·04	0·13		0·37	0·28	0·46	
	Indonesia	0·37	0·32	0·41		0·04	-0·03	0·11		0·70	0·63	0·78	
	Japan	0·18	0·15	0·22		-0·05	-0·12	0·01		-0·07	-0·13	-0·01	
	Korea	0·04	0·02	0·06		0·02	-0·02	0·06		-0·01	-0·05	0·03	
	Macao	0·35	0·30	0·41		0·02	-0·07	0·12		0·50	0·40	0·59	
	Malaysia	0·38	0·33	0·43		0·10	0·02	0·18		0·77	0·69	0·86	
	New Zealand	0·22	0·17	0·28		0·01	-0·09	0·10		0·49	0·39	0·59	
	Philippines	0·28	0·21	0·34		0·46	0·36	0·57		1·14	1·05	1·23	
	Singapore	0·37	0·33	0·41		0·17	0·10	0·24		0·54	0·46	0·61	
	Thailand	0·48	0·43	0·52		0·25	0·17	0·32		1·06	0·99	1·13	
	Sub-total	0·23	0·22	0·24	98·2	0·08	0·06	0·10	91·9	0·39	0·37	0·41	99·3
Americas	Argentina	0·18	0·13	0·23		0·22	0·14	0·30		0·41	0·32	0·50	
	Brazil	0·27	0·22	0·32		0·15	0·06	0·23		0·55	0·46	0·64	
	Canada	0·14	0·11	0·16		0·03	-0·02	0·07		0·48	0·44	0·53	
	Chile	0·19	0·14	0·24		0·05	-0·04	0·14		0·44	0·35	0·54	
	Colombia	0·28	0·22	0·34		0·24	0·13	0·35		0·77	0·66	0·88	
	Costa Rica	0·03	-0·02	0·07		0·12	0·04	0·19		0·29	0·20	0·37	
	Dominican Republic	0·41	0·28	0·55		0·76	0·53	0·99		1·40	1·16	1·64	
	Mexico	0·15	0·09	0·21		0·14	0·04	0·25		0·53	0·42	0·64	
	Panama	0·27	0·16	0·37		0·32	0·13	0·51		0·78	0·59	0·96	
	Peru	0·22	0·14	0·29		0·29	0·08	0·49		0·61	0·43	0·80	
	United States	0·05	0·00	0·11		0·12	0·03	0·20		0·42	0·34	0·50	
	Uruguay	0·24	0·17	0·31		0·14	0·02	0·25		0·78	0·64	0·92	
	Sub-total	0·16	0·15	0·18	90·1	0·12	0·09	0·14	84·3	0·50	0·48	0·53	92·0
Europe A	Austria	0·22	0·17	0·27		0·08	0·00	0·16		0·45	0·36	0·53	
	Belgium	0·05	0·02	0·09		-0·01	-0·06	0·05		0·25	0·19	0·31	
	Croatia	0·16	0·12	0·21		-0·01	-0·08	0·07		0·35	0·27	0·42	
	Czech Republic	0·18	0·13	0·23		-0·04	-0·12	0·04		0·45	0·36	0·53	
	Denmark	0·12	0·08	0·16		0·02	-0·04	0·08		0·27	0·21	0·33	
	Finland	0·14	0·09	0·18		0·01	-0·07	0·08		0·17	0·10	0·25	
	France	0·06	0·02	0·11		0·16	0·08	0·23		0·42	0·34	0·51	
	Germany	0·13	0·06	0·19		0·10	-0·02	0·21		0·43	0·30	0·56	
	Greece	0·31	0·26	0·36		0·12	0·04	0·21		0·62	0·53	0·71	
	Iceland	0·08	0·02	0·14		0·01	-0·09	0·10		0·39	0·29	0·50	
	Ireland	0·16	0·11	0·21		-0·01	-0·10	0·07		0·23	0·14	0·32	
	Italy	0·25	0·21	0·29		0·05	-0·01	0·12		0·67	0·60	0·74	
	Luxembourg	0·16	0·11	0·21		0·19	0·10	0·27		0·63	0·54	0·73	
	Malta	0·54	0·46	0·62		-0·11	-0·25	0·03		0·82	0·66	0·97	
	Netherlands	0·07	0·04	0·11		-0·04	-0·10	0·03		0·16	0·08	0·24	
	Norway	0·10	0·06	0·15		0·00	-0·08	0·07		0·36	0·28	0·44	
	Portugal	0·09	0·04	0·13		0·14	0·07	0·22		0·50	0·42	0·58	
	Slovenia	0·29	0·24	0·34		0·06	-0·02	0·14		0·54	0·47	0·62	
	Sweden	0·12	0·08	0·17		0·02	-0·06	0·10		0·33	0·24	0·41	
	Switzerland	0·15	0·09	0·21		0·09	-0·02	0·19		0·55	0·46	0·65	
	United Kingdom	0·12	0·09	0·15		0·01	-0·05	0·06		0·25	0·19	0·30	
	Sub-total	0·15	0·14	0·16	92·7	0·04	0·02	0·05	65·4	0·39	0^·^37	0·40	93·5
Europe B/C	Albania	0·35	0·30	0·40		0·01	-0·07	0·09		0·73	0·64	0·81	
	Belarus	0·15	0·11	0·20		0·23	0·16	0·31		0·40	0·32	0·48	
	Bosnia and Herzegovina	0·26	0·21	0·32		0·15	0·06	0·25		0·59	0·49	0·69	
	Bulgaria	0·38	0·30	0·45		0·11	-0·02	0·24		0·76	0·63	0·88	
	Estonia	0·23	0·18	0·28		0·11	0·03	0·19		0·36	0·28	0·44	
	Georgia	0·32	0·26	0·38		-0·01	-0·11	0·09		0·67	0·56	0·78	
	Hungary	0·22	0·16	0·28		0·17	0·07	0·26		0·62	0·52	0·72	
	Kazakhstan	0·43	0·39	0·46		0·14	0·08	0·20		0·88	0·82	0·95	
	Kosovo	0·39	0·33	0·44		0·01	-0·10	0·11		0·77	0·67	0·88	
	Latvia	0·25	0·20	0·31		0·13	0·03	0·23		0·77	0·68	0·87	
	Lithuania	0·30	0·25	0·36		0·13	0·04	0·22		0·85	0·77	0·94	
	Moldova	0·04	-0·01	0·09		0·20	0·13	0·28		0·39	0·31	0·47	
	Montenegro	0·31	0·26	0·37		-0·12	-0·22	-0·03		0·58	0·48	0·68	
	Poland	0·23	0·17	0·28		-0·01	-0·10	0·07		0·43	0·34	0·52	
	Romania	0·35	0·29	0·41		0·30	0·20	0·40		0·55	0·45	0·65	
	Russian Federation	0·27	0·22	0·32		-0·04	-0·13	0·05		0·52	0·43	0·61	
	Serbia	0·32	0·26	0·38		0·17	0·07	0·26		0·74	0·63	0·85	
	Slovak Republic	0·20	0·14	0·26		0·11	0·01	0·21		0·61	0·51	0·70	
	Turkey	0·43	0·38	0·48		0·20	0·12	0·28		0·55	0·47	0·62	
	Ukraine	0·24	0·19	0·29		0·17	0·08	0·26		0·43	0·34	0·52	
	Sub-total	0·28	0·27	0·29	93·5	0·11	0·09	0·13	80·8	0·61	0·59	0·63	93·0
	Overall	0·23	0·22	0·24	97·6	0·09	0·08	0·10	85·3	0·49	0·48	0·50	97·8

Overall estimates of mean differences for standardised total victimised score, calculated with meta-analysis using random effects, are shown.

Comparison of subtype scores revealed that gender inequality in the total victimised score was driven by physically and verbally victimised scores (0·27, 0·27–0·28; 0·26, 0·26–0·27, respectively, Table S4). In all countries except for the Philippines, the inequality in the relational victimised score was smaller than in other subtypes (0·09, 0·08–0·09 for overall). Moreover, in half of the participating countries, gender inequality for relational victimisation was not significant or even reversed (i.e., girls more likely to be victimised).

### 3.3 Inequalities by wealth

**Figure 3:**
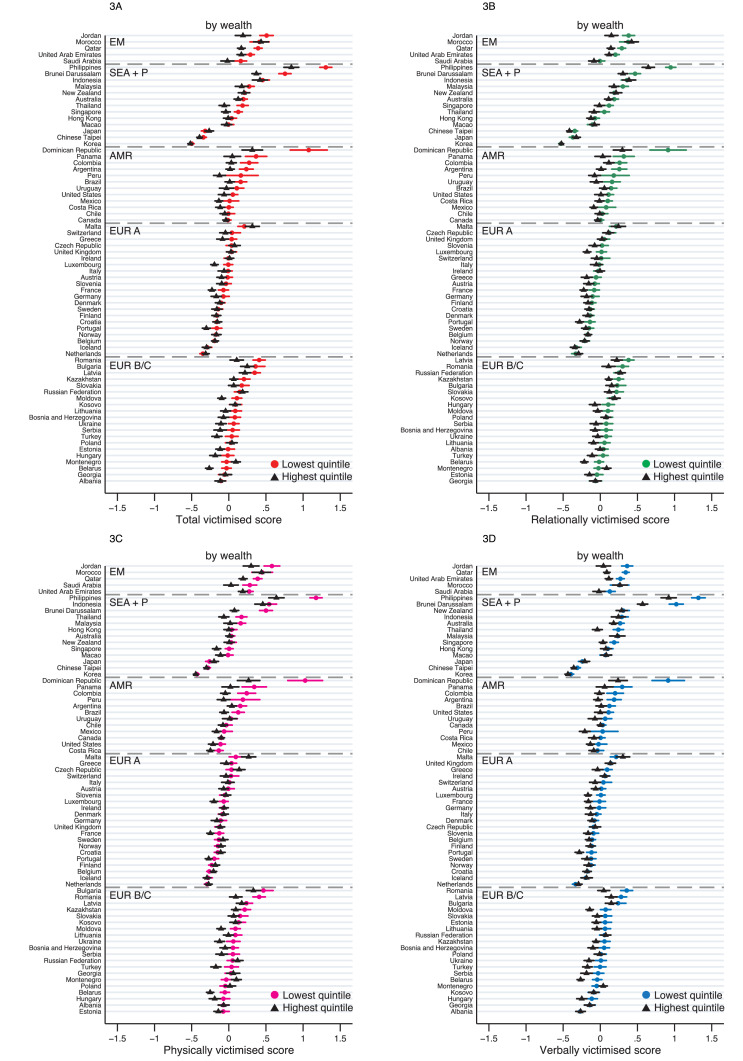
**Inequalities by wealth for victimisation scores, by country.** EM = Eastern Mediterranean; SEA+P = South East Asia & Pacific; AMR = Americas; EUR A = Europe A; EUR B/C = Europe B/C. Weighted means (in z-scores) for the total and subtypes of victimisation scores are plotted for the highest and lowest wealth for each country. Error bars show 95% CIs.

Inequalities by wealth for the total victimised score were smaller in magnitude compared to other exposures (lowest vs highest 0·09, 0·08–0·10, Figure 3 and Table 2), with considerable heterogeneity (I^2^ = 85·3). The largest inequality by wealth was observed in the Dominican Republic (0·76, 0·53–0·99) followed by the Philippines (0·46, 0·36–0·57), yet in half of the SE Asian + Pacific and three-quarters of Europe A countries, wealth inequality was not evident. Analysis by subtypes of victimisation revealed similar results (Table S5).

### 3.4 Inequalities by academic performance

**Figure 4:**
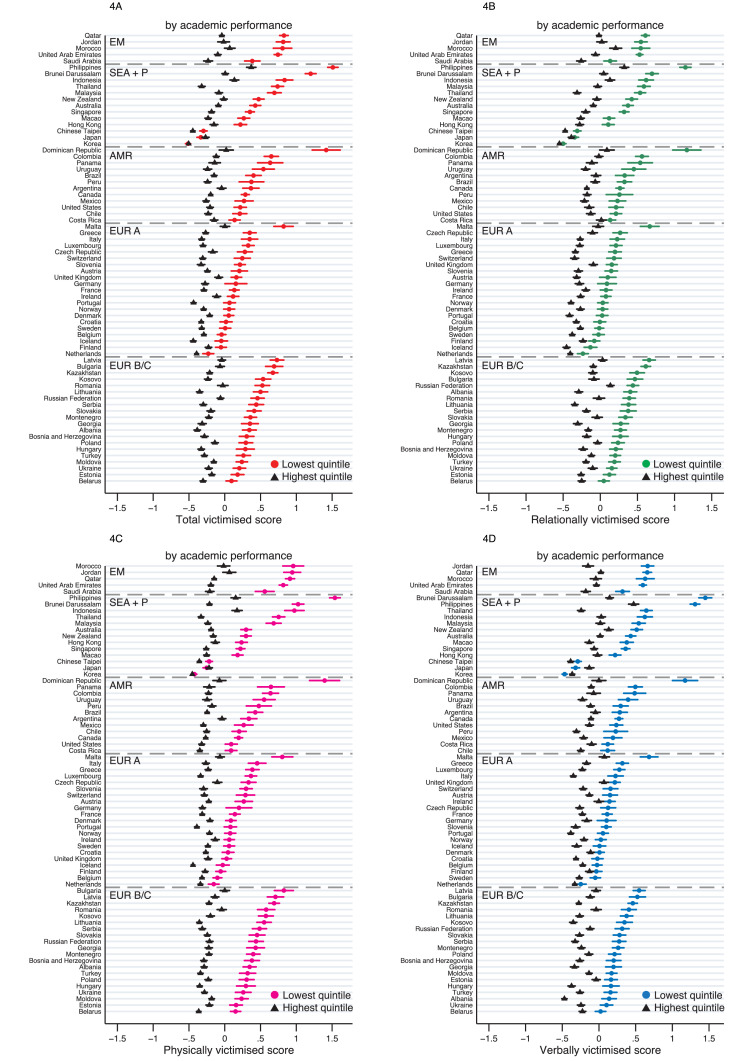
**Inequalities by academic performance for victimisation scores, by country.** EM = Eastern Mediterranean; SEA+P = South East Asia & Pacific; AMR = Americas; EUR A = Europe A; EUR B/C = Europe B/C. Weighted means (in z-scores) for the total and subtypes of victimisation scores are plotted for the highest and lowest academic performance quintiles for each country. Error bars show 95% CIs.

Inequality by academic performance was evident in almost all countries (lowest vs highest across all countries: 0·49, 0·48–0·50, Figure 4 and Table 2), though again there was substantial heterogeneity in its magnitude across countries (I^2^ = 97·8). The only exception was in Korea, where it was null, and in Japan, where it was reversed (i.e. those with higher academic performance were more likely to be victimised: -0·07, -0·13– -0·01). Analysis by subtypes revealed that this different association found in Japan and Korea was primarily driven by the reverse association for verbally victimised score (-0·19, -0·25– -0·12 for Japan; -0·10, -0·15– -0·06 for Korea, Table S6).

## 4 Discussion

This study estimated the prevalence of victimisation (overall and subtypes of relational, physical, and verbal victimisation) and inequalities by gender, wealth and academic performance across 71 high and middle-income countries. Around one-third of the 15-year-olds experienced monthly victimisation over the last 12 months at the global level, with substantial heterogeneity between countries examined, ranging from 9·3% in Korea to 64·8% in the Philippines. Analysis by subtypes of victimisation revealed large heterogeneity across the 71 countries examined. Overall, physical victimisation was least common across all countries (15·2%, range 1·6–40·9%), with varying prevalences of relational and verbal victimisation, each affecting around one-fifth of surveyed students (range from 2·6–45·4% for relational and 7·0–55·6% for verbal). These analyses again highlight large heterogeneity in victimisation subtypes across the 71 countries examined. Inequalities by wealth were considerably weaker than those for gender or academic performance. On average, boys, students with the lowest family wealth and lowest academic performance were more likely to be victimised. However, there were considerable between-country differences in the extent of observed inequalities.

Although there was a substantial between-country difference, the overall prevalence of victimisation in our study was 30·4%. This is higher compared to that previously reported in a sample of European and North American countries [6], however, our estimate corresponds with that for the Global School-based Student Health Survey, which included low-middle income countries (30·4%, defined as 1–2 days during the past 30 days) [16]. Several reasons may explain the different results across studies as well as the high between-country heterogeneity observed. First, a previous study reported that international differences in victimisation prevalence were associated with country wealth [20], which may have influenced the result. Second, the observed difference could be reflecting differences in cultural and social norms across the countries, which may influence the level of social acceptability of victimisation and its subtypes, as reported in studies of intimate partner violence [21]. Lastly, although the PISA 2018 victimisation questions are strengthened by using behavioural anchors (i.e., examples of victimisation rather than using the term ‘victimisation’), other methodological issues such as differences in the definition of each native term could also have influenced the result [22], [23], [24]. Relatedly, there may be differences in response styles of the students across countries (e.g., country differences in the magnitude or direction of social desirability bias), which may have influenced the results [25]. Although our study is not designed to answer these hypotheses, further research to explain the significant heterogeneity observed in the previous and current cross-country studies will offer additional evidence to improve our understanding of the nature of adolescent victimisation and strategies to mitigate this.

For the first time, this study investigated subtypes of victimisation across a wide range of countries, finding substantial differences across countries. For example, in some countries, rates of all three subtypes were similar (e.g., in Jordan, these ranged between 24·6 and 27·6%). In contrast, other countries showed dominant subtypes of victimisation (e.g., Japan, where 6·8% relational, 6·8% physical and 14·0% verbal). In line with previous research across 10 European countries,[26] we find that physical victimisation is less prevalent than relational and verbal victimisation across most examined countries. Our result suggests that the victimisation experiences, including the subtypes of 15-year-olds, can vary by country and support the importance of measuring victimisation subtypes in research and when considering evidence for preventive measures [26]. Furthermore, previous studies from Europe and North America suggest that different subtypes of victimisation might be associated with different sequelae; for instance, physical and relational victimisation may increase the risk of suicidal behaviour [26,27]. Future studies should explore whether differential outcomes are observed across different cultural or country settings to understand the implications of different forms of victimisation in different contexts.

In our study, boys were more likely to report victimisation in almost all countries across different regions and cultures. Previous findings on gender differences for overall victimisation have mainly been mixed [6,9,10]. likely reflecting, to some extent, differences in definition and measurement of victimisation [22], [23], [24]. Analysis by subtypes of victimisation showed similar patterns, except for relational victimisation where gender differences were smaller, a finding in line with previous research from European countries [26]. Our result suggests that the observed different association with gender by subtypes of victimisation may be more universal than previously realised.

We found a small but significant effect of wealth on victimisation, supporting a recent meta-analysis reporting a weak association between socioeconomic status and victimisation [28]. However, there was also a substantial between-country heterogeneity: while in some countries there was substantial inequality by wealth (e.g., the Dominican Republic), nearly half of the countries from Asia and three-quarters of those from Europe A, showed no wealth inequality, demonstrating that the degree to which lower socioeconomic position is a risk factor for victimisation differs by the context. The lack of inequality by socioeconomic position in victimisation experiences in some countries may reflect different social norms around the socioeconomic position, or it could result from successful anti-bullying policies in these countries.

In contrast to the inequality by wealth, the inequality by academic performance on victimisation was large and evident across almost all countries and subtypes of victimisation. The result is in line with previous research, which mainly included countries from Northern America and Europe and reported an association between victimisation and lower academic performance [13]. A notable exception to this finding was observed in Japan and Korea, where students with higher academic performance were more likely to be victimised; this difference was primarily driven by the higher risk of verbal victimisation among those with higher academic performance. The findings suggest different associations between academic performance and victimisation in certain Asian countries. Although our study cannot provide an explanation for the underlying mechanism for this reversed association observed in Japan and Korea, these results, along with that for wealth highlight that risk factors for victimisation are context-dependent and that any anti-bullying policies or interventions implemented should consider the context in their country.

The strength of this study is the use of a nationally representative sample of 15-year-olds across 71 high and middle-income countries from different cultures and regions. We were able to examine the role of both individual and contextual predictors, including academic performance. Victimisation experiences in PISA 2018 were measured using behavioural anchors instead of the term bullying, helping minimise the effect of cultural differences in the definition of bullying [24]. We were also able to provide the prevalence and correlates by different subtypes of victimisation.

Nonetheless, our study also has some limitations. First, due to the cross-sectional design of the study, causality and directions of associations could not be addressed (e.g., for academic performance). Second, as the PISA sample is a nationally representative sample of 15-year-olds enrolled in education, young people not in schooling were not included. Relatedly, subtypes of victimisation are known to change with the developmental stage (e.g., physical victimisation is more common in early childhood and relational victimisation becomes more common in later childhood) [6]. Whether these developmental changes occur similarly across different cultural contexts is unknown; therefore, examining the prevalences and associated risk factors across countries at different developmental stages will help further unpack the findings observed in the current study. Our sample bias analysis revealed that boys, those from lower wealth and lower academic performance, were more likely to be excluded from our study due to non-response on the bullying measure, which may have led to underestimating the associations observed in our study. Although PISA 2018 includes over 70 countries, some countries with large adolescent populations, such as India or countries from the African region, were not included in the PISA 2018. Likewise, low-income countries were not included, limiting the scope of country contexts examined. It will be crucial for future cross-national studies to include these countries, not least as many adolescents globally live in these countries. Third, although PISA 2018 used various behavioural anchors to measure victimisation, other types of victimisation not listed may remain undetected. Our focus on frequent victimisation (i.e., more than a few times a month) may have excluded students who experienced infrequent but more severe victimisation [29]. However, it is worth noting that different types of victimisation may cluster [30], and frequent victimisation are reported to have a more detrimental effect on mental health [16]. Fourth, PISA 2018 only assessed victimisation; however, it is also important to understand risk factors for students who engage in bullying behaviour or are both bullies and victims across countries. Finally, we only examined three major risk factors for victimisation in our study; however, there are other important risk factors, including but not limited to race/ethnicity, disability or gender identity, which all requires future research.

Given the lasting negative consequences of victimisation, the high prevalences reported highlight the global need for greater efforts to develop and implement preventative strategies. The significant heterogeneity in both prevalence and inequalities across countries requires further research as they should prove beneficial in identifying successful strategies to reduce the prevalence of victimisation and the multiple forms of inequality observed. Designing and implementation efforts may be most likely to succeed when taking into account the predominant types of victimisation and supporting those who are particularly vulnerable, as observed in each country.

## Declaration of Competing Interest

All authors declare no competing interests.

## Appendix Supplementary materials

Image, application 1

Image, application 2
