# Supplementary material for: Bullying victimisation in adolescence: prevalence and inequalities by gender, socioeconomic status and academic performance across 71 countries
Source: eClinicalMedicine. 2021 Oct 11;41:101142. doi: 10.1016/j.eclinm.2021.101142 (PMC8517283; doi:10.1016/j.eclinm.2021.101142)
Supplement: Supplementary file 1 [file mmc1.docx]

**Supplemental Document S1 Details of victimisation variables**

**Supplemental Table S1 Sample bias analysis**

**Supplemental Table S2 Correlation between victimisation variables**

**Supplemental Table S3 Demographic characteristics by country**

**Supplemental Table S4 Overall estimate of *inequalities by gender*, by subtype of victimised scores**

**Supplemental Table S5 Overall estimate of *inequalities by wealth*, by subtype of victimised scores**

**Supplemental Table S6 Overall estimate of *inequalities by academic performance*, by subtype of victimised scores**

**Figure S1 Sample selection flow-chart**
