# Supplementary material for: Bullying victimisation in adolescence: prevalence and inequalities by gender, socioeconomic status and academic performance across 71 countries
Source: eClinicalMedicine. 2021 Oct 11;41:101142. doi: 10.1016/j.eclinm.2021.101142 (PMC8517283; doi:10.1016/j.eclinm.2021.101142)
Supplement: Supplementary file 2 [file mmc2.pdf]

Hosozawa et al. Bullying victimisation in adolescence: prevalence and inequalities by gender, socioeconomic status and academic performance across 71 countries

Supplementary materials captions

Supplemental Document S1 Details of victimisation variables 2

Supplemental Table S1 Sample bias analysis 3

Supplemental Table S2 Correlation between victimisation variables 4

Supplemental Table S3 Demographic characteristics by country 5

Supplemental Table S4 Overall estimate of *inequalities by gender,* by subtype of victimised scores 8

Supplemental Table S5 Overall estimate of *inequalities by wealth,* by subtype of victimised scores 11

Supplemental Table S6 Overall estimate of *inequalities by academic performance,* by subtype of victimised scores 14

Figure S1 Sample selection flow-chart 17

Supplemental Document S1 Details of victimisation variables

Questionnaire items		Items used for victimisation variables by subtype
'During the past 12 months, how often have you had the following experiences in school? (Some experiences can also happen in social media.)' (response categories: 1= never or almost never, 2 = a few times a year, 3 = a few times a month, and 4 = once a week or more)		
1.	Other students left me out of things on purpose.	Relational
2.	Other students made fun of me.	Verbal
3.	I was threatened by other students.	Verbal
4.	Other students took away or destroyed things that belonged to me.	Physical
5.	I got hit or pushed around by other students.	Physical
6.	Other students spread nasty rumours about me.	Relational

To quantify the prevalence for overall victimisation and by subtypes (relational, physical and verbal), we created dichotomised variables representing being victimised more than a few times a month in either of the six (for overall victimisation experiences) or two (for subtypes) victimisation questions.

In addition, we created a composite score for overall victimisation and by subtypes to represent the frequency of victimisation by summing these questions (i.e. total victimised score, relationally victimised score, physically victimised score and verbally victimised score).

Supplemental Table S1 Sample bias analysis

	Total sample for included countries (N = 526,161)				*p* value for group difference
	Analytic sample (n =421,437)		Non-analytic sample (n = 104,724)		
	n	%	n	%	
Boys	208,179	49·5	55,890	51·9	< 0·0001
Wealth quintile					
1 (lowest)	78,143	19·1	22,850	26·1	< 0·0001
2	82,347	19·3	19,550	21·9	
3	84,962	20·3	17,855	18·9	
4	86,904	20·6	16,727	17·2	
5 (highest)	89,081	20·8	16,071	16·0	
Academic performance quintile					
1 (lowest)	72,311	17·9	31,086	28·9	< 0·0001
2	81,478	19·6	22,502	21·6	
3	84,655	20·2	19,696	19·4	
4	87,569	20·7	17,480	17·2	
5 (highest)	95,424	21·7	13,960	12·9	

Note: Unweighted numbers and weighted percentages are shown. N varies due to missing data.

Supplemental Table S2 Correlation between victimisation variables

	Mean	SD	1	2	3
1. Total victimised score	8·88	3·95			
2. Relationally victimised score	3·06	1·47	0·89****		
3. Physically victimised score	2·78	1·43	0·89****	0·67****	
4. Verbally victimised score	3·04	1·50	0·91****	0·73****	0·73****

Note: Raw scores and weighted correlations are shown. $^{****}p$ <0.0001

Supplemental Table S3 Demographic characteristics by country

Region	Country	Total N	Boys		Wealth[a]		Academic performance[b]	
			n	%	Mean	SD	Mean	SD
Eastern Mediterranean	Jordan	7,929	3,794	48·54	-1·00	1·21	-1·80	3·76
	Morocco	2,455	1,351	55·10	-1·56	1·34	-3·81	3·50
	Qatar	11,557	5,329	46·99	0·41	1·31	-1·56	4·85
	Saudi Arabia	5,290	2,615	49·69	-0·03	1·22	-3·52	3·69
	United Arab Emirates	17,124	8,326	46·69	0·52	1·37	-0·74	5·04
	Sub-total	44,355	21,415	50·45	-0·49	1·45	-3·03	3·94
SE Asian + Pacific	Australia	10,640	5,424	51·46	0·67	0·85	2·52	4·83
	Brunei Darussalam	4,739	2,376	50·18	-0·11	1·26	-1·22	4·85
	Chinese Taipei	7,067	3,513	49·96	-0·50	0·87	3·24	4·82
	Hong Kong	5,597	2,825	50·89	-0·46	0·84	3·96	4·39
	Indonesia	11,604	5,597	48·75	-2·33	1·09	-3·81	3·43
	Japan	5,946	2,919	48·97	-0·41	0·78	3·41	4·40
	Korea	6,593	3,415	52·22	-0·45	0·56	3·38	4·73
	Macao	3,757	1,903	50·65	-0·54	0·86	4·52	3·96
	Malaysia	5,805	2,842	48·90	-1·36	1·13	-1·27	3·94
	New Zealand	5,050	2,398	48·90	0·45	0·91	2·75	4·79
	Philippines	6,281	2,952	47·42	-2·14	1·36	-5·36	3·76
	Singapore	6,417	3,281	51·25	0·01	0·83	5·27	4·79
	Thailand	8,351	3,806	46·58	-1·31	1·05	-2·26	3·83
	Sub-total	87,847	43,251	48·76	-1·64	1·38	-1·92	5·09
Americas	Argentina	7,809	3,756	49·40	-0·98	0·96	-2·65	4·16
	Brazil	6,475	3,129	49·11	-1·23	1·01	-2·11	4·44
	Canada	18,998	9,252	49·04	0·49	0·99	3·59	4·33
	Chile	5,037	2,506	50·53	-0·74	0·95	-0·79	4·09
	Colombia	5,238	2,592	49·52	-1·48	1·30	-1·95	3·97
	Costa Rica	6,295	3,090	48·48	-1·10	1·16	-1·89	3·52
	Dominican Republic	1,454	750	51·67	-1·37	1·24	-5·18	3·79
	Mexico	3,886	1,908	49·21	-1·18	1·30	-1·52	3·77
	Panama	1,778	930	51·85	-1·31	1·60	-4·06	4·01
	Peru	2,180	1,201	55·26	-1·39	1·26	-1·18	4·05
	United States	4,573	2,290	50·26	0·45	1·04	2·22	4·78
	Uruguay	2,955	1,359	45·69	-1·04	1·00	-0·80	4·12
	Sub-total	66,678	32,763	49·90	-0·30	1·37	0·37	4·98
	Austria	5,329	2,626	48·93	0·16	0·82	2·17	4·56

Europe A	Belgium	7,249	3,553	49·11	0·19	0·82	2·78	4·58
	Croatia	5,529	2,722	49·07	-0·41	0·67	1·03	4·15
	Czech Republic	6,184	3,074	50·76	-0·10	0·83	2·27	4·47
	Denmark	6,046	2,999	49·54	0·99	0·66	2·66	4·14
	Finland	5,047	2,526	49·99	0·21	0·74	3·40	4·31
	France	4,762	2,396	49·96	-0·01	0·86	2·42	4·54
	Germany	2,339	1,239	53·12	0·19	0·88	3·21	4·86
	Greece	5,548	2,734	49·42	-0·32	0·81	0·11	4·17
	Iceland	2,451	1,217	49·48	0·45	0·83	1·41	4·52
	Ireland	4,535	2,259	49·34	0·20	0·80	2·79	4·11
	Italy	8,755	4,506	51·37	-0·11	0·68	1·33	4·41
	Luxembourg	4,582	2,323	51·00	0·37	1·03	1·45	4·83
	Malta	2,789	1,355	48·58	0·44	0·84	0·91	4·90
	Netherlands	3,621	1,812	49·34	0·47	0·72	3·43	4·49
	Norway	5,294	2,595	49·12	0·67	0·89	2·47	4·57
	Portugal	4,898	2,458	50·35	-0·08	0·83	2·05	4·51
	Slovenia	5,001	2,629	50·31	0·01	0·66	2·64	4·25
	Sweden	4,805	2,342	48·90	0·46	0·88	2·81	4·57
	Switzerland	3,598	1,909	53·29	0·18	0·84	2·22	4·80
	United Kingdom	12,215	5,912	47·76	0·45	0·95	2·81	4·56
	Sub-total	110,577	55,186	50·01	0·16	0·88	2·42	4·59
Europe B/C	Albania	5,986	2,931	49·77	-1·27	1·09	-1·73	3·55
	Belarus	5,391	2,783	51·39	-0·79	0·69	1·03	4·21
	Bosnia and Herzegovina	5,414	2,723	49·95	-0·62	0·72	-2·42	3·62
	Bulgaria	3,741	1,863	50·98	-0·34	0·95	-1·02	4·61
	Estonia	4,826	2,387	49·49	-0·10	0·70	3·78	4·11
	Georgia	4,369	2,100	48·30	-1·07	0·93	-3·07	3·82
	Hungary	4,293	2,083	48·82	-0·23	0·82	1·47	4·47
	Kazakhstan	15,729	2,156	50·68	-1·07	0·82	-2·51	3·56
	Kosovo	4,346	7,885	48·24	-0·89	0·91	-4·64	3·19
	Latvia	4,542	2,203	48·28	-0·37	0·79	1·76	3·97
	Lithuania	5,540	2,789	50·55	-0·31	0·79	1·21	4·35
	Moldova	4,889	2,423	49·70	-1·18	0·95	-1·38	4·19
	Montenegro	5,525	2,699	49·54	-0·51	0·94	-1·27	3·77
	Poland	5,062	2,458	49·38	-0·16	0·71	3·11	4·40
	Romania	4,485	2,318	51·41	-0·62	0·91	-1·28	4·42
	Russian Federation	6,463	3,107	48·40	-0·55	0·84	1·54	3·97
	Serbia	4,866	2,363	49·03	-0·63	0·68	-0·06	4·39
	Slovak Republic	4,834	2,393	49·37	-0·22	0·81	1·07	4·46

Turkey	6,627	3,322	49·83	-1·33	0·96	0·42	4·12
Ukraine	5,052	2,578	51·14	-0·87	0·80	0·65	4·29
Sub-total	111,980	55,564	49·48	-0·78	0·95	0·76	4·35
Overall	421,437	208,179	49·45	-0·81	1·44	-0·24	5·01

Note: Unweighted numbers and weighted means and percentages are shown. SD = standard deviation.

[a] Wealth was derived from student reported family wealth possessions, a continuous variable estimated using item response theory scaling by the OECD.

[b] Academic performance score was derived from predicted values of the confirmatory factor analysis, which used plausible values from three academic domains (mathematics, reading and science) measured in PISA 2018.

Supplemental Table S4 Overall estimate of *inequalities by gender,* by subtype of victimised scores

Region	Country	Relationally victimised score				Physically victimised score				Verbally victimised score			
		Mean difference[a]	95% CI		I^2	Mean difference[a]	95% CI		I^2	Mean difference[a]	95% CI		I^2
Eastern Mediterranean	Jordan	0·44	0·39	0·49		0·73	0·67	0·78		0·58	0·53	0·63	
	Morocco	0·24	0·15	0·32		0·53	0·43	0·62		0·36	0·27	0·44	
	Qatar	0·38	0·34	0·41		0·55	0·51	0·60		0·56	0·52	0·60	
	Saudi Arabia	0·32	0·27	0·37		0·56	0·51	0·62		0·52	0·47	0·57	
	United Arab Emirates	0·40	0·37	0·43		0·61	0·58	0·64		0·59	0·56	0·63	
	Sub-total	0·38	0·36	0·40	83·9	0·60	0·58	0·62	86·2	0·56	0·54	0·58	86·0
SE Asian + Pacific	Australia	-0·04	-0·08	0·00		0·34	0·31	0·38		0·29	0·25	0·34	
	Brunei Darussalam	0·23	0·17	0·29		0·50	0·43	0·56		0·50	0·42	0·57	
	Chinese Taipei	0·11	0·08	0·14		0·16	0·14	0·19		0·19	0·16	0·22	
	Hong Kong	0·27	0·22	0·32		0·40	0·35	0·45		0·41	0·36	0·46	
	Indonesia	0·25	0·21	0·29		0·38	0·33	0·42		0·35	0·30	0·39	
	Japan	0·02	-0·02	0·05		0·25	0·21	0·28		0·22	0·18	0·26	
	Korea	-0·04	-0·06	-0·02		0·05	0·03	0·07		0·09	0·06	0·12	
	Macao	0·22	0·17	0·28		0·34	0·28	0·39		0·38	0·32	0·45	
	Malaysia	0·24	0·18	0·29		0·36	0·31	0·41		0·41	0·35	0·46	
	New Zealand	-0·09	-0·15	-0·03		0·36	0·31	0·41		0·33	0·27	0·39	
	Philippines	0·22	0·16	0·28		0·33	0·26	0·40		0·20	0·13	0·26	
	Singapore	0·17	0·13	0·22		0·37	0·33	0·41		0·44	0·40	0·49	
	Thailand	0·40	0·36	0·45		0·45	0·41	0·50		0·41	0·37	0·46	
	Sub-total	0·10	0·09	0·12	98·1	0·23	0·22	0·24	98·5	0·28	0·27	0·29	96·9
Americas	Argentina	0·02	-0·03	0·06		0·25	0·21	0·30		0·21	0·16	0·25	
	Brazil	0·16	0·11	0·22		0·29	0·24	0·33		0·27	0·22	0·32	
	Canada	-0·06	-0·09	-0·04		0·23	0·21	0·26		0·20	0·17	0·23	
	Chile	0·07	0·02	0·13		0·20	0·15	0·24		0·24	0·19	0·29	
	Colombia	0·16	0·11	0·22		0·32	0·26	0·38		0·26	0·20	0·31	
	Costa Rica	-0·11	-0·16	-0·06		0·14	0·10	0·18		0·06	0·01	0·10	
	Dominican Republic	0·24	0·11	0·37		0·43	0·30	0·57		0·43	0·31	0·56	
	Mexico	0·00	-0·06	0·06		0·23	0·17	0·29		0·18	0·13	0·24	
	Panama	0·16	0·06	0·26		0·33	0·23	0·43		0·21	0·11	0·32	
	Peru	0·12	0·05	0·20		0·24	0·17	0·32		0·21	0·14	0·28	
	United States	-0·11	-0·17	-0·06		0·16	0·12	0·21		0·10	0·05	0·16	
	Uruguay	0·09	0·02	0·16		0·32	0·25	0·39		0·22	0·15	0·28	
	Sub-total	0·01	-0·01	0·03	93·5	0·23	0·22	0·24	83·7	0·20	0·18	0·21	85·2
	Austria	0·03	-0·02	0·08		0·33	0·28	0·37		0·24	0·19	0·29	

Europe A	Belgium	-0·13	-0·17	-0·10		0·17	0·14	0·20		0·11	0·08	0·15	
	Croatia	-0·02	-0·07	0·03		0·28	0·23	0·32		0·19	0·14	0·23	
	Czech Republic	0·00	-0·06	0·05		0·31	0·26	0·36		0·18	0·13	0·23	
	Denmark	-0·06	-0·10	-0·02		0·21	0·17	0·25		0·18	0·14	0·22	
	Finland	-0·08	-0·12	-0·03		0·28	0·24	0·32		0·17	0·12	0·22	
	France	-0·04	-0·09	0·01		0·15	0·11	0·20		0·06	0·01	0·11	
	Germany	-0·07	-0·14	0·00		0·24	0·18	0·31		0·17	0·11	0·24	
	Greece	0·15	0·10	0·20		0·34	0·29	0·39		0·33	0·28	0·38	
	Iceland	-0·06	-0·12	0·00		0·15	0·10	0·20		0·13	0·07	0·20	
	Ireland	-0·09	-0·15	-0·04		0·28	0·23	0·33		0·24	0·18	0·29	
	Italy	0·10	0·06	0·14		0·32	0·28	0·36		0·24	0·21	0·28	
	Luxembourg	0·02	-0·03	0·07		0·24	0·19	0·29		0·17	0·12	0·22	
	Malta	0·26	0·18	0·34		0·64	0·56	0·72		0·54	0·46	0·62	
	Netherlands	-0·12	-0·16	-0·07		0·19	0·15	0·23		0·13	0·09	0·17	
	Norway	-0·08	-0·12	-0·03		0·18	0·14	0·22		0·18	0·13	0·22	
	Portugal	-0·01	-0·05	0·04		0·13	0·09	0·17		0·10	0·06	0·14	
	Slovenia	0·08	0·02	0·13		0·41	0·36	0·46		0·28	0·24	0·33	
	Sweden	-0·09	-0·13	-0·04		0·26	0·21	0·31		0·16	0·11	0·20	
	Switzerland	-0·02	-0·08	0·04		0·24	0·19	0·30		0·17	0·12	0·23	
	United Kingdom	-0·09	-0·13	-0·06		0·22	0·19	0·25		0·20	0·16	0·23	
	Sub-total	-0·03	-0·04	-0·02	92·0	0·24	0·23	0·25	93·3	0·19	0·18	0·20	90·4
Europe B/C	Albania	0·28	0·23	0·33		0·32	0·27	0·37		0·34	0·29	0·38	
	Belarus	0·05	0·01	0·10		0·18	0·14	0·23		0·17	0·12	0·21	
	Bosnia and Herzegovina	0·13	0·07	0·18		0·32	0·26	0·37		0·25	0·20	0·31	
	Bulgaria	0·20	0·13	0·28		0·44	0·36	0·52		0·36	0·29	0·43	
	Estonia	0·04	-0·01	0·09		0·29	0·24	0·34		0·28	0·22	0·33	
	Georgia	0·24	0·18	0·30		0·33	0·27	0·39		0·29	0·23	0·34	
	Hungary	0·05	-0·01	0·11		0·29	0·23	0·34		0·25	0·20	0·31	
	Kazakhstan	0·33	0·29	0·36		0·43	0·39	0·46		0·38	0·35	0·41	
	Kosovo	0·27	0·22	0·33		0·36	0·29	0·42		0·40	0·34	0·45	
	Latvia	0·11	0·05	0·17		0·31	0·25	0·37		0·25	0·19	0·31	
	Lithuania	0·19	0·14	0·24		0·36	0·30	0·41		0·26	0·21	0·32	
	Moldova	-0·04	-0·09	0·01		0·06	0·01	0·11		0·09	0·04	0·14	
	Montenegro	0·19	0·13	0·24		0·34	0·28	0·39		0·31	0·26	0·37	
	Poland	0·07	0·01	0·12		0·29	0·24	0·35		0·24	0·19	0·30	
	Romania	0·22	0·16	0·28		0·37	0·31	0·44		0·33	0·27	0·39	
	Russian Federation	0·17	0·12	0·22		0·28	0·23	0·34		0·27	0·21	0·32	
	Serbia	0·17	0·11	0·23		0·37	0·31	0·43		0·32	0·26	0·37	
	Slovak Republic	0·03	-0·03	0·09		0·31	0·25	0·37		0·21	0·15	0·26	

	Turkey	0·28	0·23	0·33		0·44	0·39	0·48		0·43	0·39	0·48	
	Ukraine	0·10	0·05	0·15		0·26	0·21	0·30		0·27	0·22	0·32	
	Sub-total	0·16	0·15	0·17	94·2	0·31	0·30	0·32	92·1	0·29	0·28	0·30	90·8
	Overall	0·09	0·08	0·09	97·7	0·27	0·27	0·28	97·3	0·26	0·26	0·27	96·7

Note: Overall estimates were calculated with meta-analysis using random effects. [a] Mean differences for boys-girls are shown.

Supplemental Table S5 Overall estimate of *inequalities by wealth,* by subtype of victimised scores

Region	Country	Relationally victimised score				Physically victimised score				Verbally victimised score			
		Mean Difference[a]	95% CI		I^2	Mean Difference[a]	95% CI		I^2	Mean Difference[a]	95% CI		I^2
Eastern Mediterranean	Jordan	0·23	0·15	0·31		0·28	0·19	0·37		0·32	0·24	0·40	
	Morocco	-0·05	-0·19	0·10		0·01	-0·16	0·17		-0·01	-0·15	0·14	
	Qatar	0·15	0·08	0·21		0·20	0·13	0·27		0·26	0·19	0·32	
	Saudi Arabia	0·08	0·00	0·16		0·25	0·15	0·35		0·15	0·06	0·24	
	United Arab Emirates	0·08	0·03	0·14		0·09	0·03	0·14		0·15	0·10	0·21	
	Sub-total	0·12	0·09	0·15	75·1	0·16	0·13	0·20	80·9	0·20	0·17	0·23	83·0
SE Asian + Pacific	Australia	0·08	0·01	0·14		0·02	-0·04	0·08		0·09	0·02	0·16	
	Brunei Darussalam	0·16	0·07	0·26		0·43	0·32	0·53		0·45	0·34	0·57	
	Chinese Taipei	0·07	0·02	0·12		0·02	-0·02	0·07		0·05	0·00	0·10	
	Hong Kong	0·06	-0·03	0·14		0·04	-0·05	0·13		0·03	-0·06	0·11	
	Indonesia	-0·03	-0·09	0·04		0·09	0·01	0·16		0·05	-0·02	0·11	
	Japan	-0·05	-0·11	0·01		-0·06	-0·13	0·00		-0·03	-0·10	0·04	
	Korea	-0·01	-0·05	0·04		0·02	-0·02	0·05		0·04	-0·01	0·09	
	Macao	-0·03	-0·12	0·06		0·10	0·01	0·19		-0·01	-0·11	0·09	
	Malaysia	0·12	0·04	0·20		0·14	0·06	0·22		0·01	-0·08	0·09	
	New Zealand	-0·02	-0·12	0·07		0·03	-0·06	0·11		0·02	-0·08	0·12	
	Philippines	0·30	0·21	0·40		0·53	0·43	0·64		0·40	0·30	0·50	
	Singapore	0·13	0·06	0·21		0·17	0·10	0·24		0·15	0·08	0·23	
	Thailand	0·14	0·07	0·21		0·24	0·16	0·31		0·28	0·21	0·36	
	Sub-total	0·05	0·03	0·07	83·7	0·08	0·06	0·09	93·5	0·09	0·07	0·11	90·9
Americas	Argentina	0·24	0·16	0·33		0·11	0·04	0·19		0·22	0·14	0·30	
	Brazil	0·09	0·01	0·17		0·19	0·11	0·27		0·11	0·03	0·20	
	Canada	0·04	-0·01	0·08		0·00	-0·04	0·04		0·03	-0·02	0·07	
	Chile	0·03	-0·05	0·12		0·04	-0·04	0·13		0·05	-0·03	0·14	
	Colombia	0·14	0·04	0·25		0·29	0·18	0·39		0·21	0·11	0·31	
	Costa Rica	0·11	0·03	0·19		0·11	0·05	0·18		0·09	0·01	0·17	
	Dominican Republic	0·62	0·40	0·84		0·76	0·54	0·99		0·68	0·46	0·89	
	Mexico	0·17	0·06	0·28		0·10	0·00	0·20		0·11	0·01	0·21	
	Panama	0·28	0·11	0·46		0·32	0·14	0·51		0·24	0·05	0·42	
	Peru	0·26	0·07	0·45		0·26	0·06	0·46		0·24	0·05	0·43	
	United States	0·10	0·01	0·20		0·10	0·02	0·18		0·11	0·02	0·21	
	Uruguay	0·21	0·10	0·33		0·02	-0·10	0·13		0·14	0·03	0·25	
	Sub-total	0·11	0·09	0·14	80·0	0·09	0·07	0·11	87·1	0·11	0·08	0·13	80·6
	Austria	0·08	0·00	0·16		0·06	-0·02	0·15		0·08	0·00	0·15	

Europe A	Belgium	0·00	-0·07	0·06		-0·06	-0·11	-0·01		0·04	-0·02	0·10	
	Croatia	0·02	-0·06	0·09		-0·04	-0·11	0·04		0·01	-0·07	0·08	
	Czech Republic	0·01	-0·07	0·10		-0·10	-0·19	-0·02		-0·02	-0·10	0·06	
	Denmark	0·03	-0·03	0·09		-0·02	-0·08	0·05		0·03	-0·03	0·09	
	Finland	0·05	-0·03	0·13		-0·05	-0·12	0·02		0·02	-0·06	0·09	
	France	0·14	0·06	0·22		0·12	0·05	0·19		0·15	0·07	0·23	
	Germany	0·08	-0·03	0·19		0·05	-0·06	0·16		0·12	0·00	0·23	
	Greece	0·13	0·04	0·21		0·07	-0·02	0·16		0·13	0·05	0·21	
	Iceland	0·02	-0·08	0·11		0·01	-0·08	0·10		-0·01	-0·11	0·10	
	Ireland	-0·02	-0·11	0·06		-0·01	-0·09	0·07		0·01	-0·08	0·10	
	Italy	0·04	-0·03	0·11		0·02	-0·05	0·09		0·08	0·02	0·15	
	Luxembourg	0·19	0·11	0·28		0·13	0·05	0·21		0·17	0·09	0·26	
	Malta	-0·03	-0·17	0·11		-0·17	-0·31	-0·04		-0·09	-0·23	0·05	
	Netherlands	-0·04	-0·11	0·03		-0·02	-0·08	0·05		-0·04	-0·11	0·02	
	Norway	0·00	-0·07	0·08		-0·04	-0·11	0·03		0·03	-0·05	0·10	
	Portugal	0·14	0·06	0·22		0·07	0·00	0·14		0·16	0·09	0·24	
	Slovenia	0·10	0·01	0·19		-0·02	-0·10	0·07		0·07	-0·01	0·15	
	Sweden	0·04	-0·04	0·13		-0·05	-0·13	0·03		0·06	-0·02	0·13	
	Switzerland	0·06	-0·05	0·16		0·07	-0·04	0·17		0·11	0·01	0·21	
	United Kingdom	0·03	-0·03	0·09		-0·01	-0·06	0·04		0·00	-0·06	0·06	
	Sub-total	0·05	0·03	0·06	53·3	-0·00	-0·02	0·02	64·7	0·05	0·03	0·07	63·0
Europe B/C	Albania	0·05	-0·04	0·12		0·00	-0·08	0·08		0·00	-0·08	0·07	
	Belarus	0·20	0·12	0·28		0·20	0·13	0·27		0·22	0·15	0·30	
	Bosnia and Herzegovina	0·15	0·06	0·24		0·11	0·02	0·20		0·15	0·06	0·24	
	Bulgaria	0·08	-0·05	0·20		0·13	0·00	0·27		0·09	-0·03	0·21	
	Estonia	0·10	0·02	0·18		0·07	-0·01	0·14		0·12	0·03	0·20	
	Georgia	0·00	-0·10	0·10		-0·03	-0·13	0·07		0·00	-0·09	0·09	
	Hungary	0·18	0·08	0·28		0·12	0·03	0·21		0·14	0·05	0·22	
	Kazakhstan	0·13	0·08	0·19		0·12	0·06	0·18		0·12	0·06	0·17	
	Kosovo	-0·02	-0·12	0·08		0·04	-0·07	0·15		0·00	-0·10	0·10	
	Latvia	0·16	0·06	0·26		0·06	-0·04	0·16		0·13	0·04	0·23	
	Lithuania	0·15	0·06	0·23		0·09	0·00	0·18		0·11	0·02	0·20	
	Moldova	0·14	0·06	0·22		0·19	0·11	0·27		0·22	0·14	0·30	
	Montenegro	-0·11	-0·20	-0·02		-0·14	-0·23	-0·04		-0·09	-0·18	0·00	
	Poland	0·01	-0·08	0·10		-0·06	-0·15	0·02		0·02	-0·07	0·10	
	Romania	0·19	0·09	0·28		0·31	0·21	0·42		0·31	0·21	0·41	
	Russian Federation	-0·02	-0·11	0·07		-0·07	-0·16	0·02		-0·02	-0·10	0·07	
	Serbia	0·14	0·04	0·23		0·15	0·06	0·25		0·16	0·07	0·25	
	Slovak Republic	0·10	0·00	0·20		0·09	-0·02	0·19		0·11	0·02	0·20	

	Turkey	0·15	0·07	0·23		0·21	0·14	0·29		0·17	0·10	0·25	
	Ukraine	0·12	0·03	0·20		0·19	0·10	0·27		0·16	0·07	0·25	
	Sub-total	0·10	0·08	0·12	70·0	0·10	0·08	0·11	82·8	0·11	0·09	0·13	78·5
	Overall	0·08	0·07	0·08	75·8	0·06	0·05	0·07	87·6	0·09	0·08	0·10	83·5

Note: Overall estimates were calculated with meta-analysis using random effects. [a] Mean differences for lowest-highest quintiles are shown.

Supplemental Table S6 Overall estimate of *inequalities by academic performance,* by subtype of victimised scores

Region	Country	Relationally victimised score				Physically victimised score				Verbally victimised score			
		Mean Difference[a]	95%CI		I^2	Mean Difference[a]	95%CI		I^2	Mean Difference[a]	95%CI		I^2
Eastern Mediterranean	Jordan	0·53	0·45	0·61		0·88	0·79	0·97		0·81	0·73	0·89	
	Morocco	0·34	0·20	0·48		0·97	0·82	1·13		0·67	0·54	0·81	
	Qatar	0·63	0·56	0·69		1·06	0·99	1·13		0·63	0·56	0·70	
	Saudi Arabia	0·39	0·31	0·47		0·77	0·67	0·87		0·50	0·41	0·59	
	United Arab Emirates	0·59	0·54	0·65		1·01	0·96	1·07		0·63	0·58	0·69	
	Sub-total	0·54	0·51	0·58	87·2	0·97	0·93	1·01	85·8	0·65	0·61	0·68	85·1
SE Asian + Pacific	Australia	0·47	0·40	0·53		0·49	0·43	0·55		0·42	0·35	0·49	
	Brunei Darussalam	0·65	0·56	0·74		1·24	1·14	1·34		1·30	1·20	1·41	
	Chinese Taipei	0·16	0·11	0·22		0·14	0·09	0·18		0·10	0·05	0·15	
	Hong Kong	0·39	0·30	0·47		0·37	0·28	0·46		0·24	0·15	0·33	
	Indonesia	0·49	0·42	0·56		0·80	0·72	0·88		0·59	0·52	0·67	
	Japan	0·04	-0·02	0·10		-0·03	-0·09	0·03		-0·19	-0·25	-0·12	
	Korea	0·05	0·01	0·09		0·03	0·00	0·06		-0·10	-0·15	-0·06	
	Macao	0·38	0·29	0·47		0·43	0·34	0·52		0·51	0·41	0·62	
	Malaysia	0·62	0·54	0·71		0·92	0·83	1·00		0·52	0·44	0·61	
	New Zealand	0·47	0·37	0·56		0·46	0·37	0·55		0·38	0·28	0·48	
	Philippines	0·82	0·73	0·91		1·39	1·30	1·48		0·84	0·75	0·93	
	Singapore	0·52	0·44	0·59		0·48	0·41	0·55		0·43	0·35	0·51	
	Thailand	0·85	0·78	0·92		1·09	1·01	1·16		0·89	0·82	0·96	
	Sub-total	0·35	0·33	0·37	98·5	0·37	0·35	0·38	99·5	0·33	0·31	0·35	99·2
Americas	Argentina	0·39	0·30	0·47		0·37	0·29	0·46		0·33	0·25	0·42	
	Brazil	0·39	0·31	0·48		0·67	0·58	0·76		0·41	0·32	0·50	
	Canada	0·45	0·40	0·49		0·46	0·42	0·50		0·38	0·34	0·43	
	Chile	0·36	0·27	0·45		0·45	0·36	0·54		0·37	0·28	0·46	
	Colombia	0·58	0·48	0·68		0·87	0·76	0·98		0·60	0·50	0·71	
	Costa Rica	0·12	0·03	0·21		0·44	0·36	0·51		0·22	0·13	0·31	
	Dominican Republic	1·08	0·85	1·31		1·47	1·23	1·71		1·17	0·95	1·40	
	Mexico	0·45	0·34	0·55		0·57	0·46	0·67		0·40	0·30	0·51	
	Panama	0·65	0·48	0·82		0·86	0·68	1·04		0·56	0·38	0·74	
	Peru	0·44	0·27	0·61		0·66	0·46	0·85		0·54	0·36	0·71	
	United States	0·34	0·25	0·43		0·41	0·34	0·49		0·37	0·28	0·45	
	Uruguay	0·65	0·51	0·79		0·80	0·65	0·94		0·62	0·49	0·75	
	Sub-total	0·42	0·39	0·45	89·9	0·52	0·50	0·55	93·6	0·41	0·38	0·43	88·7
	Austria	0·42	0·34	0·50		0·49	0·40	0·58		0·28	0·20	0·37	

Europe A	Belgium	0·26	0·19	0·32		0·22	0·16	0·28		0·20	0·13	0·26	
	Croatia	0·32	0·24	0·39		0·31	0·23	0·39		0·29	0·22	0·37	
	Czech Republic	0·37	0·29	0·46		0·44	0·35	0·53		0·39	0·31	0·47	
	Denmark	0·30	0·23	0·36		0·29	0·23	0·36		0·12	0·06	0·19	
	Finland	0·15	0·07	0·23		0·22	0·14	0·29		0·09	0·01	0·17	
	France	0·34	0·26	0·43		0·46	0·37	0·54		0·34	0·25	0·42	
	Germany	0·37	0·25	0·50		0·51	0·38	0·64		0·27	0·14	0·40	
	Greece	0·53	0·45	0·62		0·62	0·52	0·71		0·49	0·40	0·58	
	Iceland	0·32	0·22	0·42		0·42	0·32	0·51		0·31	0·20	0·42	
	Ireland	0·27	0·19	0·36		0·20	0·11	0·28		0·15	0·05	0·24	
	Italy	0·50	0·43	0·57		0·72	0·64	0·79		0·58	0·51	0·64	
	Luxembourg	0·49	0·40	0·58		0·70	0·61	0·80		0·50	0·41	0·60	
	Malta	0·69	0·55	0·84		0·87	0·71	1·03		0·61	0·46	0·76	
	Netherlands	0·17	0·09	0·25		0·19	0·11	0·27		0·08	0·00	0·17	
	Norway	0·43	0·35	0·51		0·29	0·21	0·37		0·23	0·15	0·31	
	Portugal	0·44	0·36	0·52		0·47	0·39	0·55		0·44	0·36	0·52	
	Slovenia	0·44	0·36	0·52		0·60	0·51	0·68		0·42	0·34	0·49	
	Sweden	0·36	0·27	0·44		0·30	0·21	0·38		0·22	0·14	0·30	
	Switzerland	0·53	0·44	0·62		0·58	0·48	0·67		0·37	0·28	0·46	
	United Kingdom	0·25	0·19	0·31		0·26	0·21	0·31		0·15	0·09	0·21	
	Sub-total	0·36	0·34	0·38	87·8	0·38	0·37	0·40	94·6	0·29	0·27	0·31	92·8
Europe B/C	Albania	0·69	0·61	0·78		0·64	0·55	0·72		0·61	0·53	0·69	
	Belarus	0·30	0·22	0·38		0·52	0·44	0·59		0·25	0·17	0·32	
	Bosnia and Herzegovina	0·44	0·35	0·53		0·67	0·57	0·77		0·46	0·37	0·55	
	Bulgaria	0·55	0·43	0·67		0·83	0·70	0·96		0·64	0·53	0·76	
	Estonia	0·38	0·30	0·46		0·37	0·29	0·46		0·20	0·11	0·29	
	Georgia	0·58	0·47	0·69		0·65	0·54	0·77		0·53	0·43	0·63	
	Hungary	0·45	0·35	0·56		0·65	0·55	0·75		0·54	0·44	0·63	
	Kazakhstan	0·71	0·65	0·77		0·91	0·85	0·97		0·74	0·68	0·79	
	Kosovo	0·60	0·50	0·69		0·77	0·66	0·89		0·70	0·60	0·80	
	Latvia	0·63	0·53	0·72		0·85	0·75	0·94		0·59	0·49	0·68	
	Lithuania	0·72	0·64	0·80		0·90	0·82	0·99		0·64	0·56	0·72	
	Moldova	0·32	0·24	0·40		0·42	0·33	0·50		0·31	0·22	0·39	
	Montenegro	0·44	0·34	0·53		0·62	0·52	0·72		0·50	0·41	0·60	
	Poland	0·28	0·19	0·36		0·54	0·45	0·63		0·35	0·26	0·44	
	Romania	0·41	0·31	0·50		0·62	0·52	0·72		0·45	0·35	0·54	
	Russian Federation	0·30	0·22	0·39		0·65	0·56	0·74		0·44	0·35	0·52	
	Serbia	0·56	0·46	0·67		0·80	0·69	0·91		0·60	0·50	0·70	
	Slovak Republic	0·38	0·29	0·48		0·70	0·60	0·79		0·55	0·46	0·63	

Turkey	0·38	0·31	0·46		0·66	0·58	0·73		0·42	0·34	0·49	
Ukraine	0·26	0·17	0·34		0·54	0·45	0·63		0·35	0·26	0·43	
Sub-total	0·48	0·46	0·50	92·7	0·66	0·64	0·68	91·6	0·49	0·48	0·51	92·4
Overall	0·41	0·40	0·42	95·5	0·50	0·49	0·51	98·4	0·40	0·39	0·41	97·3

Note: Overall estimates were calculated with meta-analysis using random effects. [a] Mean differences for lowest-highest quintiles are shown.

Figure S1 Sample selection flow-chart

Figure S1

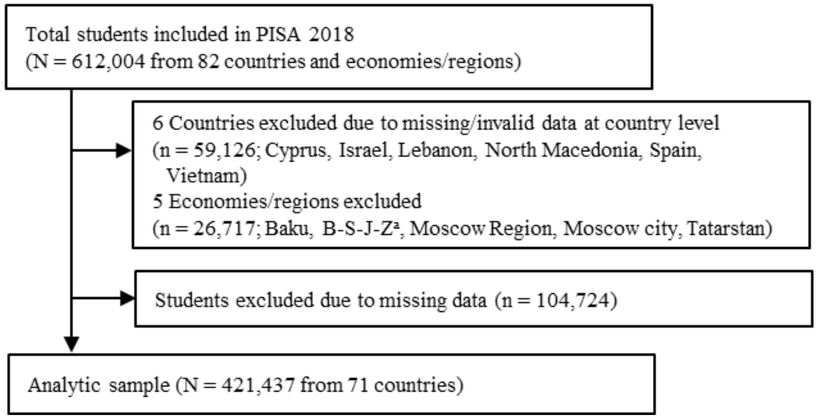

A flow-chart for sample selection. [a] B-S-J-Z= Beijing, Shanghai, Jiangsu, and Zheijiang
